# Comparison of dienogest or combinations with ethinylestradiol/estradiol valerate on the pain score of women with endometriosis: A prospective cohort study

**DOI:** 10.1097/MD.0000000000038585

**Published:** 2024-07-05

**Authors:** Aslihan Yurtkal, Mahmut Oncul

**Affiliations:** a Faculty of Medicine, Department of Gynecology and Obstetrics, Kafkas University, Kars, Turkey; b Faculty of Medicine, Department of Gynecology and Obstetrics, İstanbul University Cerrahpaşa, Istanbul, Turkey.

**Keywords:** Anti-Mullerian hormone, cancer antigen-125, dienogest, endometrioma, endometriosis, lifelong treatment, low-cost treatment, oral hormonal contraceptives, pelvic pain, visual analog score

## Abstract

Endometriosis is one of the most frequent gynecologic disorders. The pathognomonic symptom of endometriosis is pelvic pain. The recommended pain medications are oral hormonal contraceptives, progestin therapy, danazol, gonadotropin-releasing hormone analogs, nonsteroidal anti-inflammatory drugs, and aromatase inhibitors. In this study, we aimed to compare the efficiency of costing dienogest (DNG) and low-cost oral contraceptives regarding visual analog scores (VAS) score of pelvic pain and also cancer antigen-125 (CA-125), anti-Mullerian hormone (AMH) levels, and size of endometrioma in the patients with endometriosis which is a chronic disease that requires a lifelong management plan. In our study, 18 to 45-year-old patients presented to our institution’s gynecology and obstetrician department for various complaints over 2 years, and endometriosis diagnoses were included. Patients were divided into 3 groups (20 patients in each medication group) according to the given medication: cyclic DNG (Visanne) or 0.03 mg ethinylestradiol combined with 2 mg DNG (Dienille) or estradiol valerate combined with 2 mg DNG (Qlarista). We recorded all patients’ CA-125/AMH values and VAS scores of pelvic pain. All patients gave informed consent. There was no statistically significant difference between pre-medication and post-medication levels of CA-125, AMH, VAS score, and cyst size in all groups. However, statistically, significant decreases were seen in the cyst size and VAS score, indicating response to therapy in all groups. In conclusion, we think it is more reasonable to use cost-effective oral contraceptive medications, which also cause common side effects, instead of costing DNG since all drugs have the same efficiency and success.

## 1. Introduction

Endometriosis is a chronic inflammatory condition that is prevalent, nonmalignant, and dependent on estrogen. Women in various hormonal phases, including premenarchal, reproductive, and postmenopausal stages, can be affected by the impact of endometriosis.^[1]^ While the distinctive indicator of the ailment is pain, the symptoms vary widely. Dysmenorrhea, persistent pelvic discomfort, and dyspareunia are common manifestations of endometriosis-associated pain. This condition affects approximately 5% of females in the reproductive age group.^[2]^ In patient groups experiencing infertility and pelvic pain complaints, the incidence can rise from 30% to −50%. The concept of treatment burden is crucial in chronic illnesses such as endometriosis. Various components, including administered treatments, management of side effects, gynecological appointments, imaging techniques, recurring tests, past surgical experiences, long-time follow-ups, lifestyle adjustments, treatment budget, and vice versa concealed costs, collectively form the economic impact of endometriosis. The combined weight of the ailment and its treatment can profoundly affect patients and their families. Endometriosis is acknowledged as a persistent condition requiring continuous treatment throughout one’s life. The symptoms associated with endometriosis have a significant impact on the quality of life for affected individuals, affecting their daily activities, work, education, social connections, and both mental and sexual well-being.^[3]^ Given these challenges, the choice of treatment becomes crucial. The approach to endometriosis treatment needs to be personalized.^[4]^

Given this fact, minimizing recurrent surgical interventions by optimizing medical treatment is prudent. No definitive cure exists for endometriosis, and the primary therapeutic objective is pain relief. Although the efficacy of treatment options is comparable, differences exist in patient tolerance, costs, and side effects,

Our research suggested assessing the medical management of persistent ailment using dienogest (DNG), and 2 combined oral contraceptive (COC) regimens were chosen. Pain was identified as the primary variable to gauge treatment efficacy, measured with the visual analog score (VAS). Additionally, secondary variables included the evaluation of cancer antigen-125 (CA-125), anti-Mullerian hormone (AMH), and the dimensions of endometriomas. Our study results were deliberated with a concise literature review, contributing insights into selecting drugs for primary treatment. The objective is to alleviate the cost of treatment and disease for patients and their relatives and formulate more efficient management protocols considering the national healthcare burden.

## 2. Materials and methods

Our study involved individuals aged 18 to 45 who sought care at the Gynecology outpatient clinic over 2 years, with approval from the ethics committee (10.04.2018-135383). Participants were selected following the criteria outlined by the European Society of Human Reproduction and Embryology (ESHRE) and the American Society for Reproductive Society (ASRM), based on factors such as operation pathology reports indicating endometriosis or the presence of endometrioma in evaluations using pelvic magnetic resonance imaging (MRI)/transvaginal ultrasound (TVUSG)/transabdominal ultrasound (TAUSG). Table 1 outlines the inclusion and exclusion criteria for our study. Patients receiving medical treatment were categorized into treatment groups: continuous cyclic DNG (Visanne @BAYER), 0.03 mg ethinylestradiol (EE) (Dienille @EXELTIS) combined with 2 mg DNG or estradiol valerate (Qlarista @BAYER) combined with 2 mg DNG. Pelvic pain was assessed using the VAS, while CA-125 and AMH values were documented. Six cycles after treatment, participants underwent a follow-up, during which AMH/CA-125 levels and VAS scores were updated.

**Table 1:** Inclusion and exclusion criteria.

Inclusion criteria	Exclusion criteria
Patients between the ages of 18 and 45	Patients with autoimmune diseases
Patients who have a pathology report consistent with endometriosis	Patients with malignancy
Patients with endometrioma	Patients with serious systemic diseases and high risk of morbidity
	Patients with neuropathy (considering the pain scoring)
	Patients with mental and psychiatric illness (considering the pain scoring)
	Patients with fibromyalgia (considering the pain scoring)
	Patients with chronic pain killer use (considering the pain scoring)
	Patients who have used GNRH (gonodatropin releasing hormone) analogs in the past 6 months
	Patients who have used COC in the past 3 months
	Patients with a diagnosis of deep pelvic endometriosis
	Patients with ovarian failure
	Conditions that cause an excess of CA-125
	Conditions that can cause high AMH like polycystic ovary syndrome

Notably, no heterogeneous findings suggestive of a pathologically vascularized cyst, papillary protrusion, or Doppler-detected hyperechogenicity resembling malignancy were observed in evaluating adnexal masses. Patients with elevated CA-125 levels, in whom malignancy could not be ruled out, are excluded. Our study adheres to the Declaration of Helsinki, follows the principles of Good Clinical Practice, and complies with the ethical regulations governing the research.

The sample size was calculated by G-Power 3.12; an alpha of 0.05 with 90% power gave an effect size of 0.7.^[5]^ While evaluating the findings obtained in the study, the SPSS 24.0 statistical package program was used for statistical analysis. While considering the study data, the Kolmogorov–Smirnov distribution test was used to examine the normal distribution and descriptive statistical methods (frequency, percentage, mean, standard deviation, and median). A 1-way ANOVA test was used for the intergroup comparisons of customarily distributed parameters in continuous variables. The Kruskal–Wallis test was used for intergroup comparisons of non-normally distributed parameters. Pearson Chi-Square test was used to compare qualitative data. The results were evaluated at the 95% confidence interval at the *P* < .05 significance level.

## 3. Results

Seventy-eight patients were enlisted for the exploration, conforming to the specified inclusion and exclusion criteria (Table 1).

**Figure 1.**
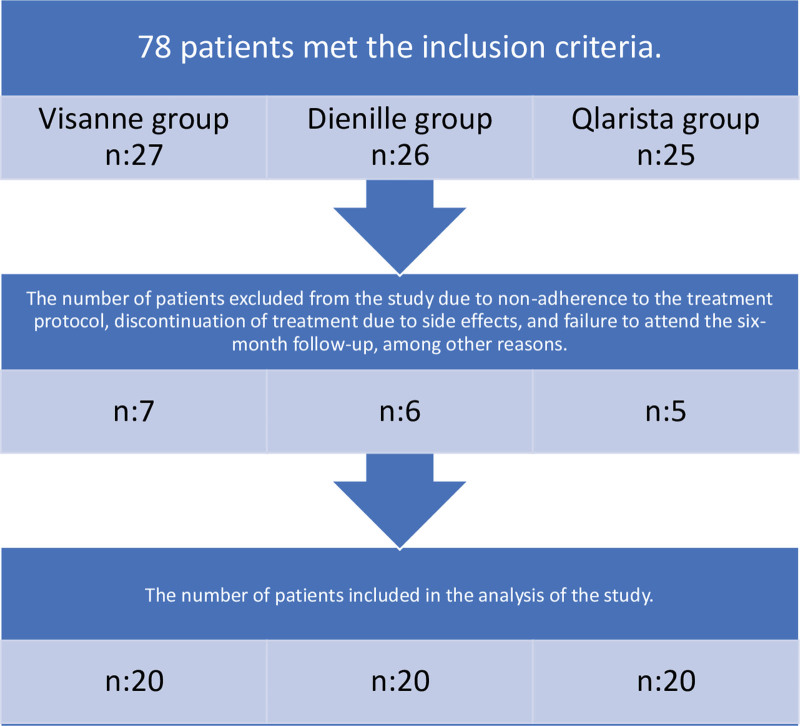
Evaluation of patients excluded from the study group.

All participating patients gave their accord. An equal distribution of 20 patients was assigned to each treatment faction, pondering the excluded participants. Details about patients not embraced in the assessment and those ousted from the exploration faction are outlined in Figure 1.

Considering the parameters specified in the table, there is no significant discrepancy between drug factions (*P* > .05) (Table 2).

**Table 2:** Demographic characteristics by drug groups.

		Visanne		Dienille		Qlarista		*P*
		n	%	n	%	n	%	
Education status	Literate	2	10.0	3	15.0	2	10.0	.285^a^
	Primary school	4	20.0	1	5.0	1	5.0	
	Middle school	3	15.0	8	40.0	3	15.0	
	High school	5	25.0	7	35.0	9	45.0	
	University	6	30.0	1	5.0	5	25.0	
Body mass index (BMI)	Weak	1	5.0	0	0.0	1	5.0	.792^a^
	Normal	10	50.0	7	35.0	6	30.0	
	Light weight	5	25.0	8	40.0	8	40.0	
	Obesity	4	20.0	5	25.0	5	25.0	
Family story	Yes	13	65.0	17	85.0	15	75.0	.344^a^
	No	7	35.0	3	15.0	5	25.0	
Smoking	Yes	6	30.0	8	40.0	6	30.0	.741^a^
	No	14	70.0	12	60.0	14	70.0	
Alcohol consumption	Yes	1	5.0	0	0.0	0	0.0	.362^a^
	No	19	95.0	20	100.0	20	100.0	
Coffee consumption	Yes	11	55.0	5	25.0	11	55.0	. 089^a^
	No	9	45.0	15	75.0	9	45.0	
Menarche age	11	4	20.0	4	20.0	4	20.0	.965^a^
	12	3	15.0	6	30.0	6	30.0	
	13	3	15.0	4	20.0	3	15.0	
	14	5	25.0	3	15.0	4	20.0	
	15	4	20.0	3	15.0	2	10.0	
	16	1	5.0	0	0.0	1	5.0	
Menstrual cycle	Irregular	9	45.0	9	45.0	11	55.0	.766^a^
	Regular	11	55.0	11	55.0	9	45.0	
Infertility	Yes	16	80.0	14	70.0	15	75.0	.766^a^
	No	4	20.0	6	30.0	5	25.0	
	Primary	4	100.0	3	50.0	3	60.0	.241^a^
	Secondary	0	0.0	3	50.0	2	40.0	
Gestational status	Nulliparity	10	50.0	7	35.0	6	30.0	.525^a^
	Multiparity	9	45.0	9	45.0	10	50.0	
	Virgo	1	5.0	4	20.0	4	20.0	
Loss of job due to pain complaint	Yes	13	65.0	12	60.0	12	60.0	.932^a^
	No	7	35.0	8	40.0	8	40.0	
Operation	Yes	13	65.0	14	70.0	12	60.0	.803^a^
	No	7	35.0	6	30.0	8	40.0	
MRI results	Yes	14	70.0	16	80.0	14	70.0	.711^a^
	No	6	30.0	4	20.0	6	30.0	
		Med	SD	Med	SD	Med	SD	.535^b^
	Menarche age	13,250	1585	12,750	1372	12,850	1496	
	Menstrual period	5550	2212	5550	2460	5250	2593	.903^b^
	Age	29,800	7252	30,600	8444	31,800	8345	.732^b^

a Chi-square test.

b One-way ANOVA.

There was no significant incongruity between the drug factions regarding the visual analog scale (VAS) at the initial examination and VAS scores in the 6th month after drug utilization (*P* > .05).

- *Visanne group:* The diminution in VAS evaluation in the 6th month after drug utilization was statistically notable (*P* < .05).
- *Qlarista group:* The diminution in VAS evaluation in the 6th month after drug utilization was statistically notable (*P* < .05)
- *Dienille group:* The diminution in VAS evaluation in the 6th month after drug utilization was statistically notable (*P* < .05) (Table 3).

**Table 3:** Changes in the evaluation of VAS according to drug groups.

	Visanne		Dienille		Qlarista		*P*
	Med	SD	Med	SD	Med	SD	
Evaluation of the VAS at the first examination	7200	2462	7850	2207	7650	2231	.660^a^
6th-month examination VAS evaluation after drug use	3300	2003	4000	2575	4600	2761	.257^a^
*P*	.000^b^		.000^b^		.000^b^		

a One-way ANOVA.

b Paired-samples *T* test.

There was no significant discrepancy between the drug factions in the initial CA-125 and post-drug CA-125 levels (*P* > .05).

- *Visanne group:* Compared to the CA-125 level at the initial examination, the diminution in the CA-125 level at the 6th-month test was statistically notable (*P* < .05).
- In the Qlarista and Dienille groups, the change in CA-125 level in the 6th month after drug utilization was not statistically notable (*P* > .05) (Table 4).

**Table 4:** Change of CA-125 levels according to drug groups.

	Visanne		Dienille		Qlarista		*P*
	Med	SD	Med	SD	Med	SD	
First examination CA-125 value	47,450	25,258	51,450	45,958	46,300	22,813	.875^a^
After medication 6th-month examination CA-125 value	32,300	14,087	51,650	88,650	42,750	38,555	.558^a^
*P*	.001^b^		.984^b^		.669^b^		

a One-way ANOVA.

b Paired-samples *T* test.

There was no significant discrepancy between the drug factions in terms of AMH at the initial examination and AMH levels in the 6th month after drug utilization (*P* > .05).

- In the Visanne, Qlarista, and Dienille groups, the initial examination was done according to the AMH level after drug utilization. The change in the AMH level at the 6th-month examination was not statistically notable (*P* > .05) (Table 5).

**Table 5:** Variation of AMH levels according to drug groups.

	Visanne		Dienille		Qlarista		*P*
	Med	SD	Med	SD	Med	SD	
First examination AMH value	2563	1267	2385	0.968	2632	1189	.782^a^
After medication 6th-month examination AMH value	2544	1277	2267	1066	2568	1209	.673^a^
*P*	.783^b^		.062^b^		.357^b^		

a One-way ANOVA

b Paired-samples *T* test.

There was no significant discrepancy between the drug factions regarding VAS at the initial examination and TVUSG findings in the 6th month after drug utilization (*P* > .05).

- *Visanne group:* The diminution in TVUSG findings in the 6th month after drug utilization was statistically notable (*P* < .05).
- *Qlarista group:* The diminution in TVUSG findings in the 6th month after drug utilization was statistically notable (*P* < .05)
- *Dienille group:* The diminution in TVUSG findings in the 6th month after drug utilization was statistically notable (*P* < .05) (Table 6).

**Table 6:** Change of TVUSG (mm^2^) findings according to drug groups.

	Visanne		Dienille		Qlarista		*P*
	Med	SD	Med	SD	Med	SD	
First examination TVUSG finding	1357	900	1669	1079	1396	1272	.620^a^
After medication 6th month examination TVUSG finding	851	1007	1320	1111	872	1138	.313^a^
*P*	.012^b^		.020^b^		.002^b^		

a Kruskal–Wallis test.

b Wilcoxon signed rank test.

## 4. Discussion

The medical management of symptomatic endometriosis aims to inhibit ovulation, reduce serum estradiol levels, and suppress uterine blood flow. Various drugs are utilized for pain relief, each with a comparable effect size, regardless of their mechanism of action. However, differences exist in accessibility, safety, tolerability, and cost. Endometriosis lacks a curative treatment, and the therapeutic duration may extend over several years.

In a literature review, Jensen et al^[6]^ conducted a comprehensive study involving 18 research articles, with 5 receiving full or partial industry support and the remaining 13 (72%) conducted by independent groups. Results indicated that both cyclic and continuous use of combined hormonal therapies significantly and effectively alleviate endometriosis-related pain. Approximately 2/3 of patients with endometriosis express satisfaction with the use of COC.

Among women with symptomatic endometriosis, Harada et al^[7]^ conducted a large, multicenter, placebo-controlled, randomized controlled trial (RCT) involving women with symptomatic endometriosis. The study demonstrated that low-dose COC treatment improves dysmenorrhea, non-menstrual pain, and profound dyspareunia. Satisfaction with estrogen-progestin treatment for symptomatic endometriosis has been supported by studies conducted between 1996 and 2017.

Harada et al^[8]^ evaluated the effects of COCs in their double-blind, placebo-controlled study. A significant decrease was detected in the evaluation of the VAS score in dysmenorrhea complaints. Non-menstrual pelvic pain scores were low, and there were differences between treatment groups at baseline. Since it is difficult to evaluate this data even in a properly designed RCT study, a clinically significant non-menstrual pain reduction with COC therapy could not be detected. Although the double-blind and placebo-controlled design of the study strengthened it, there were some limitations. Although it was designed as laparoscopic detection of endometriosis or endometrioma by imaging as diagnostic criteria for suitability, 95% of the participants included in the study were diagnosed with endometrioma detection by imaging techniques. In this case, contrary to what is expected in the general clinical routine, it may have resulted in a population selection with more advanced diseases. However, similar reductions in the size of endometriomas in the patients using COCs and in the placebo group suggest that there may be some errors in the diagnostic classification. Harada et al showed that treatment with COCs (with a regimen of 3 consecutive days of menstrual or spotting bleeding followed by 4 days without medication) resulted in fewer spotting days and more manageable bleeding periods than those using DNG.

In a study by Caruso et al,^[9]^ a combination of 30 μg EE and 2 mg DNG in oral contraceptive therapy was administered cyclically and continuously. While a more substantial decrease in the VAS was observed in the third and 6th months of continuous administration, a significant improvement in VAS was found only in the 6th month of cyclic use.

Vercellini et al concluded that administering the same dose (2 mg) of DNG as monotherapy is higher than the cost of the compound when combined with 30 μg EE in monophasic OC.

Morotti et al^[10]^ administered a continuous progestin-only pill (POP) containing 75 μg desogestrel and COCs containing desogestrel for 6 cycles to patients diagnosed with symptomatic rectovaginal endometriosis and migraine. Both treatments resulted in a reduction in chronic pelvic pain and dyspareunia, as assessed by the VAS score. However, a decrease in dysmenorrhea was observed only in the COC treatment.

However, Casper et al^[11]^ suggest that progestogens should be preferred over COC therapy in first-line therapy, considering that estrogen receptors may be overexpressed and progesterone receptors underexpressed in ectopic endometrial implants. According to the study, COC administration of COCs may negatively serve the purpose by causing progesterone resistance and estrogen dominance, resulting in the possible progression of lesions. Casper states that the amount of EE contained in POPs (20–30 μg EE), expressed as low-dose, is supraphysiological since 5 μg EE is approximately equal to 1mg micronized estradiol. Casper based his hypothesis on clinical data showing that a history of COC use may be associated with an increased risk for endometriosis in general and deep lesions in particular. In his study, Casper also emphasizes that COC therapy is off-label for treating endometriosis.

In another recent study, Taha et al^[12]^ compared DNG with commonly used low-dose COC and found no difference in efficacy.

The goal of endometriosis treatment should be pain relief. If COC cannot achieve this, the patient may be offered a different treatment. Despite progestin-based therapy, many patients may respond inadequately to COC therapy because of progestin resistance that develops with disease progression; in this regard, defining the 1/3 patient group that benefits from the suboptimal safety or tolerance profile or high-cost drug group in the step treatment needs to be revealed. Demonstration of DNG, GnRH agonist, or antagonist activity in RCT studies: it should not be considered a systematic prescription for the routine treatment of all women with symptomatic endometriosis. With the stepped treatment protocol, unnecessary prescription of these drugs to 2/3 of patients who do not need this group of drugs can be prevented.

First-line COC can be preferred in treating mild or moderate endometriosis, which we see more frequently in clinical practice. Low-cost progestin can be tried in cases where continuous COC therapy is unsuccessful or if treatment cannot be continued due to side effects. In the presence of contraindications or complete treatment failure, DNG-like progestagens or GnRH agonists or antagonists in the future may be preferred in drug incompatibility due to side effects. In advanced endometriosis, first-line treatment can be started with gestagens.

Our study has certain limitations that warrant consideration in future research. While we conducted our study as a prospective cohort study with a 6-month follow-up of patients, the lack of complete randomization in the assignment of patients to treatment groups based on social-demographic matching poses a potential weakness in our study design.

The results of the literature review and guidelines are consistent with the results of our study. The education level, body mass index (BMI), family history, habits, age profile, age at menarche, menstruation pattern, fertility status, parity, loss of job due to pain complaints, operation history, and imaging are compared, and treatment regimens almost homogeneously were determined. Considering the evaluation of pelvic pain, which is a cardinal sign, with the VAS scale, the development of the dimensions of the detected adnexal masses, CA-125 values, and AMH values. There was no statistically significant superiority of 2 mg DNG treatment over COC treatments containing the same amount of DNG and combined with estrogen, including in symptomatic patient groups. While all 3 treatment protocols caused a significant decrease in VAS score and cyst sizes, a considerable reduction in CA-125 was detected using 2 mg DNG. When the data are evaluated, since the efficacy and success of these 3 treatment protocols, which serve the primary purpose of the treatment, are the same, it seems reasonable to prefer COC treatments with a low side-effect profile and, more importantly, cost-effective instead of high-cost DNG.

In their current study, Zhang et al^[13]^ have also compared the use of DNG and COC for the prevention of endometriosis recurrence after surgery, concluding that DNG may not be cost-effective when this area is taken into consideration.

## 5. Conclusion

National Institute for Health and Care Excellence (NICE) guidelines propose the application of COC and progestagen-like hormonal therapy for suspected endometriosis, diagnosed patients, and recurrent cases. As per the NICE committee, all treatments yield a clinically substantial reduction in pain symptoms, assessed through the VAS score, compared to a placebo. Choosing hormonal therapy for pain relief in women with endometriosis aims to diminish the need for repetitive surgeries, restrict the recurrence of lesions and symptoms over time, and cut down on patient visits, tests, procedures, and overall care costs. This approach also strives to uplift the quality of life for women by alleviating anxiety and depression levels.

Treatment options with low-dose COCs and budget-friendly progestins are recommended for these objectives, accounting for minimal side effects, high patient tolerance, and cost efficiency. Transforming to cost-effective treatment protocols is advised in instances of intolerance, side effects, or treatment failure. Despite the limited data on COC therapy in the literature, various guidelines, including ESHRE, suggest COC therapy as the primary approach. GnRH treatments, in contrast to DNG-like treatments, present a cost-effective solution and furnish contraception opportunities.

Low-dose POPs and economical progestagens capably manage endometriotic lesions and symptoms, addressing approximately 2/3 of endometriosis cases, spanning deeply infiltrated forms. The stepped treatment protocol strives to thwart unnecessary prescriptions of these drugs for the 2/3 of patients who do not necessitate this category of medications. Around 1/3 of patients may demand second-line medical treatments or surgery, prompting future genetic and pharmacological studies to concentrate on enhancing outcomes for this subgroup with a poorer prognosis.

## Author contributions

**Conceptualization:** Aslihan Yurtkal.

**Data curation:** Aslihan Yurtkal.

**Formal analysis:** Aslihan Yurtkal.

**Writing – original draft:** Aslihan Yurtkal.

**Writing – review & editing:** Aslihan Yurtkal, Mahmut Oncul.

**Methodology:** Mahmut Oncul.
